# Proteomic database mining opens up avenues utilizing extracellular protein phosphorylation for novel therapeutic applications

**DOI:** 10.1186/s12967-015-0482-4

**Published:** 2015-04-19

**Authors:** Garif Yalak, Bjorn R Olsen

**Affiliations:** Department of Developmental Biology, Harvard Medical School/Harvard School of Dental Medicine, 188 Longwood Avenue, Boston, MA 02115 USA

**Keywords:** Extracellular matrix, Extracellular protein phosphorylation, Secreted kinases, Ectokinase, Exokinase, Collagens, BMPs, MMPs

## Abstract

Recent advances in extracellular signaling suggest that extracellular protein phosphorylation is a regulatory mechanism outside the cell. The list of reported active extracellular protein kinases and phosphatases is growing, and phosphorylation of an increasing number of extracellular matrix molecules and extracellular domains of trans-membrane proteins is being documented. Here, we use public proteomic databases, collagens – the major components of the extracellular matrix, extracellular signaling molecules and proteolytic enzymes as examples to assess what the roles of extracellular protein phosphorylation may be in health and disease. We propose that novel tools be developed to help assess the role of extracellular protein phosphorylation and translate the findings for biomedical applications. Furthermore, we suggest that the phosphorylation state of extracellular matrix components as well as the presence of extracellular kinases be taken into account when designing translational medical applications.

**Electronic supplementary material:**

The online version of this article (doi:10.1186/s12967-015-0482-4) contains supplementary material, which is available to authorized users.

## Introduction

Technical advances have significantly contributed to insights into the functional role of extracellular protein phosphorylation. Most recently, protein kinases such as VLK and FAM20C (also known as Golgi casein kinase), have been shown to be secreted through the classical ER-Golgi secretory pathway [1,2]. In addition, several protein kinases and phosphatases, including PKA [3,4], PKC [5,6], CKII [7], alkaline phosphatase [8], tartrate-resistant acid phosphatase (TRAP) [9] and the PTEN phosphatase [10], have been shown to accumulate in conditioned cell culture media of different cell lines [11], as well as in human plasma and/or serum samples from cancer patients [12]. Furthermore, these kinases have been shown to be functional in conditioned serum-free cell culture media [13] and/or sera [14]. At the same time, a large number of extracellular matrix and cell surface proteins and extracellular domains of trans-membrane proteins, including fibronectin [15], vitronectin [16], osteopontin [17], collagens [13], fibrinogen [18], laminin [19], CD36 [20], β-amyloid precursor protein (APP) [21], T-cell-receptor complex (TCR) [22] and many others, have been reported to be phosphorylated [23] in vitro and in vivo. At what stages in their secretory pathway such proteins may serve as substrates for kinases is not clear. In principle, phosphorylation of secretory proteins could happen prior to secretion or in the extracellular matrix (ECM). In either case, changes in phosphorylation may have important functional consequences. Alterations in phosphorylation of secreted proteins during their transit through the secretory pathway might interfere with their folding, transport, secretion, release from the cell surface or interaction with other ECM components. Phosphorylation and dephosphorylation events in the ECM may regulate the processing, assembly, degradation and binding properties of matrix proteins. In any case, the widespread phosphorylation of extracellular matrix proteins suggests that the mechanism of reversible protein phosphorylation may not only be a mechanism inside cells as widely demonstrated, but may also be a regulatory mechanism outside cells. How important such kinase activity maybe for the functions of extracellular proteins is well illustrated by evidence that mutations in secreted kinases can lead to severe disorders. For example, the Raine syndrome is caused by loss-of-function mutations in the FAM20C protein kinase [1]. Mutations leading to lethal and nonlethal forms of this disease were shown to prevent secretion and reduce the activity in the extracellular matrix [1]. It is believed that impairment of the phosphorylation state of small integrin-binding ligands, N-linked glycoproteins (SIBLINGs), accounts for the observed biomineralization defects in Raine syndrome. The sites phosphorylated by FAM20C (at the sequence motif S-x-E) bind calcium and regulate the formation of calcium phosphate-containing hydroxyapatite (HA) crystals [24]. One protein in the SIBLING group, osteopontin (OPN), inhibits HA formation, and this inhibition depends on the degree of phosphorylation [25]. In addition, the recently described secreted kinase VLK has been found to have an important role during morphogenesis [2]. Knocking Vlk out in mice leads to severe defects in morphogenesis in multiple tissues and death within a day after birth. The exact mechanism has not been identified, but the findings support the notion that secreted kinases are important in health and disease [26-29]. Understanding the mechanisms and functional roles of extracellular protein phosphorylation may open up opportunities for novel disease treatments [30]. A well-known example of the importance of the phosphorylation state of a drug for its efficiency is the Multiple Sclerosis drug Fingolimod (FTY720). The phosphorylation state of FTY720 is believed to be regulated by the ecto-phosphatase LPP3 in vivo [31].

### Secretion pathways and extracellular ATP

Since VLK and FAM20C kinases are translated with a signal peptide it is easy to understand that they can be secreted from cells into the extracellular matrix. Other members of this protein family and closely related to FAM20C, such as FAM20A, FAM20B [32], FAM198, Four-jointed box protein 1 (FJX1) [33], also contain a signal peptide and are very likely secreted [1]. In addition to these reported examples, a large number of other kinases are predicted to have a signal peptide that may be secreted [34]. Examples include the FAM69 family members or the VLK kinase (previously PKDCC) with high sequence similarity to the FAM69 family [35]. In contrast, the protein kinases PKA [36], PKC [37], CKI [38], CKII [38,39], and the alkaline phosphatases [40] which have all been found on the external surface of the cell membrane [11], do not have a signal peptide. This raises the question of how they get to their extracellular destinations. Considering the fact that well-known secreted proteins without a signal peptide, such as interleukin 1 [41], galectin 1 and 3 [42], HMGB1 [43], FGFs [44] and others [44], use unconventional secretory pathways, it is likely that kinases may use similar mechanisms. Exosomes and microvesicles are among such unconventional secretion mechanisms [44]. Protein kinases A, C and the casein kinase II have been reported to be secreted in prostasomes, secretory granules of epithelial cells of the prostate [45]. Alkaline phosphatase [46], PTEN phosphatase [47] and the tissue-nonspecific phosphatase [48] were all shown to be secreted in exosomes. These kinases and phosphatases are then associated with the outer surface of the cell membrane [37,40,49]. Their release from the cell surface into the extracellular matrix (ECM) can be induced under cell culture conditions [37,39,50].

A sufficiently high ATP concentration is essential for extracellular phosphorylation to occur. ATP concentrations in human plasma were reported in a range of 30nM-11μM in different studies and under different conditions [51]. Furthermore, extracellular ATP concentrations of 1-5μM were reported in vivo in tissues of healthy mice compared to the enhanced concentration of at least 700μM at tumor sites [52]. Several physiological and pathophysiological events are known to increase the extracellular ATP concentration, including wounds [53], inflammation [54], ischemia [55], hypoxia [56], platelet aggregation [57], sympathetic nerve stimulation [58] or cellular damage [51] (for review see [23]). In addition, a number of regulated mechanisms were shown to lead to secretion of ATP in vesicles from nerve cells, mast cells, platelets, and T cells [11,59]. Alternatively, ATP synthase was shown to generate ATP at the cell surface from cAMP and ADP [60]. Thus a large number of mechanisms have been described to lead to ATP secretion into the ECM and fulfill the precondition of ATP availability for extracellular phosphorylation to occur.

### Nearly all collagen types, the major components of the ECM, can occur in phosphorylated states as shown by mass spectrometry data

**Figure 1:**
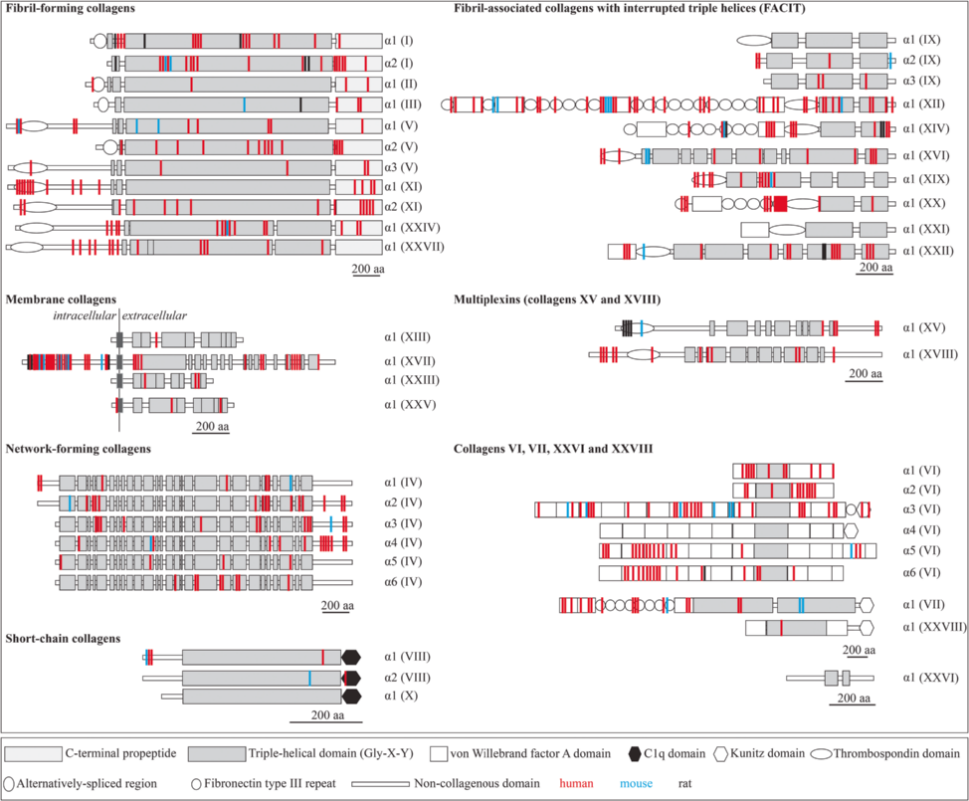
Experimentally verified phosphorylated sites for collagens. The experimentally verified phosphorylated sites were retrieved from the databases Phosida, PhosphoSitePlus, PhosphoNet, HPRD, dbPTM and UniProt. Collagen structures are represented as described [61]. The color code used to represent the position of the phosphorylated sites is red (human), blue (mouse) and black (rat).

Large scale data mining has revealed that a large number of extracellular and cell surface proteins, and extracellular domains of trans-membrane proteins can exist in phosphorylated states [23]. Among these proteins are the 28 members of the collagen protein family, the most abundant protein family in mammals and constituting up to 30% of the total protein content in the human body [61]. Mass spectrometry data for collagens show that collagen phosphorylation is much more common than previously thought; nearly all collagens can occur in phosphorylated states in different species (Figure 1, Additional file 1: Table S1). Even if only a fraction of these data can be validated, most collagens would be represented. Further support comes from curated and highly reliable databases that contain a large number of double checked phosphorylated sites in extracellular proteins [62]. The distribution of the phosphorylated residues within the collagen molecules suggests that the phosphorylation could affect a variety of specific functions. For example, a large number of phosphorylated sites are located in triple helical regions of all fibril-forming collagens. Phosphorylation at these sites may affect the polymerization of the molecules into fibrillar structures or interactions between fibrils and other extracellular matrix components. Phosphorylation of sites in the propeptide domains of procollagens could affect proteolytic cleavage of the propeptides. The extracellular domains of all membrane-associated collagens contain at least one phosphorylated site, which might be important in ECM-cell signaling or proteolytic cleavage of the surface domains. Furthermore, all network-forming and short-chain collagens [63] contain phosphorylated residues. Moreover, with the exception of collagen α1 (IX) and α1 (XXI), all fibril-associated collagens with interrupted triple helices (FACIT collagens) [64] are heavily phosphorylated. Both members of the multiplexin family of collagens (XV and XVIII) contain phosphorylated residues. Finally, except collagen chains α4 (VI), α1 (X) and α1 (XXVI), all remaining collagen chains appear to be phosphorylated. The phosphorylation map for collagens presented here is far from being complete and represents the current status of available data. A more directed study of collagen phosphorylation is likely to lead to discovery of additional phosphorylated sites in these extracellular matrix molecules.

### Phosphorylation at residues close to the N- and C-propeptide cleavage sites may affect processing of fibrillar procollagen to collagen

**Figure 2:**
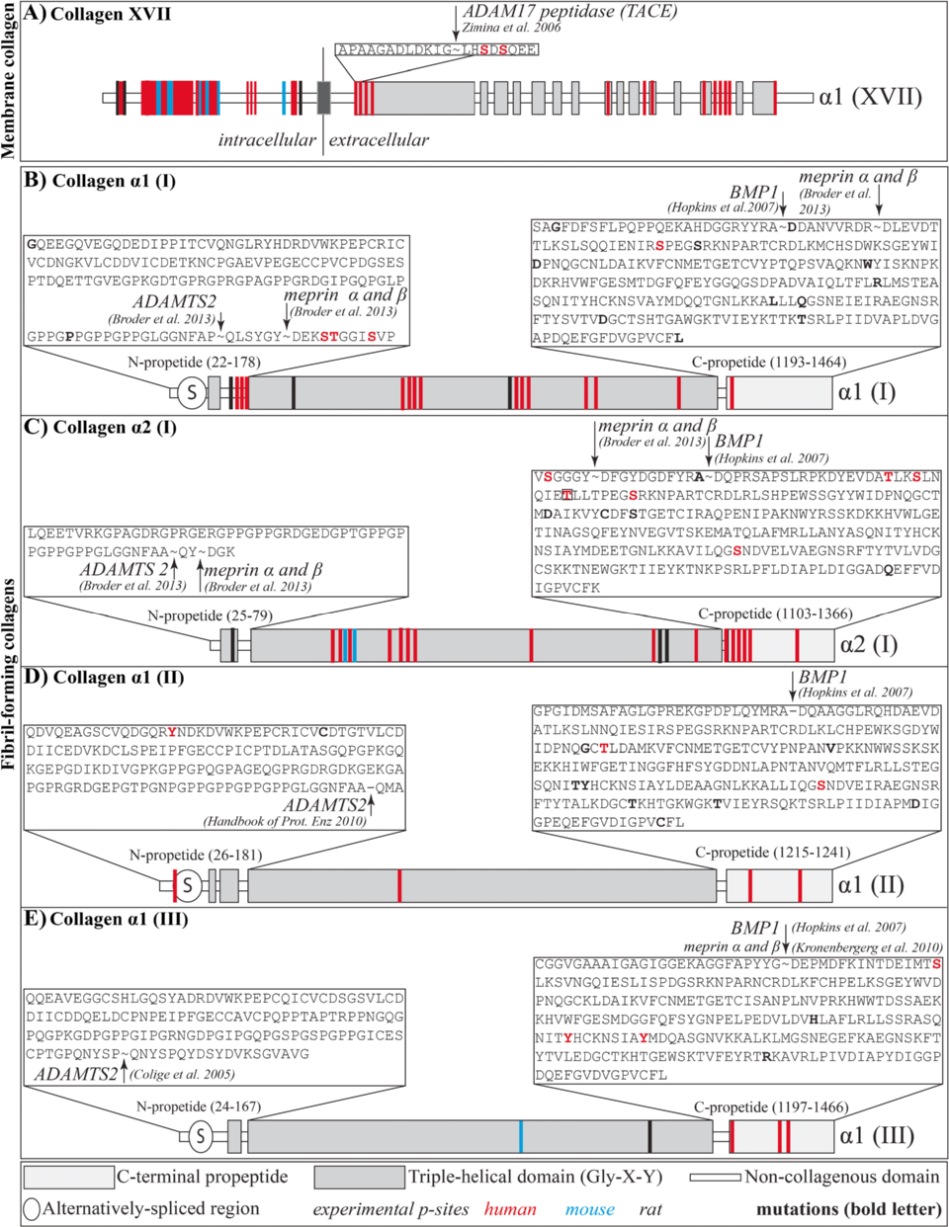
Procollagen processing by ADAMTS2, BMP1 and TACE. **A**.: Cleavage of the ecto-domain of collagen α1(XVII) by TACE. The sequence of the cleavage site of TACE (box) and the phosphorylated residues (highlighted in red) are shown. The color coding to represent the position of the phosphorylated sites used is red (human) and blue (mouse). Collagen structures are represented as described [61]. **B**-**E**.: Cleavage of the N- and C-propeptide of collagen I-III. The sequences of the N- and C-propeptide are shown in the box. Experimentally verified phosphorylated sites (red letters) and known mutations (black bold) leading to diseases are highlighted. Cleavage site of the enzymes are marked. Collagen structures are represented as described [61].

Protein phosphorylation in the ECM may well be a mechanism to regulate protein processing and degradation. Phosphorylated sites close to a cleavage site may either promote or prevent proteolytic enzyme binding. Indeed, many in vitro studies support this possibility. Whether such events happen in vivo needs to be demonstrated. Phosphorylation of vitronectin at position S362 reduces its cleavage by plasmin at the position R361-S362 [65]. Phosphorylation of complement component C3 by the surface kinase of the human parasite Leishmania, leads to more resistance to cleavage by trypsin compared with the nonphosphorylated form [66]. Phosphorylation of S8 of β-amyloid by ecto-PKA strongly inhibits its degradation by angiotensin-converting enzyme (ACE) [67]. Protein phosphorylation is also part of a mechanism that regulates shedding of transmembrane collagens from the cell surface. For example, phosphorylation of collagen XVII, a member of the family of transmembrane collagens, has been shown to regulate the proteolytic shedding of the triple-helical domain from the cell surface. Collagen XVII mediates adhesion of the epidermis to the dermis by interacting with the β4 integrin via its cytoplasmic region. The collagen XVII ectodomain is constitutively shed from the cell surface by metalloproteinases of the ADAM (a disintegrin and metalloproteinase) family. This process is inhibited by phosphorylation of collagen XVII at an extracellular site, S544, located 5 residues downstream of the TACE (ADAM17) cleavage site [13] (Figure 2A). Furthermore, sera of patients with bullous pemphigoid, a chronic autoimmune skin disease, contain autoantibodies preferentially recognizing phosphorylated collagen XVII [68].

Similarly, phosphorylation of other collagens, most notably fibril forming collagens, could be of physiological importance in the processing of procollagen to collagen by affecting cleavage of the N- and C-propeptides. Many phosphorylated sites are located close to the cleavage sites in procollagen α1 (I), α2 (I), α1 (II) and α1 (III) (Figure 2B-E). BMP1, in tandem with the procollagen C-proteinase enhancer-1 (PCPE-1), binds to the procollagens and catalyzes the proteolytic cleavage of the C-propeptide. PCPE-1 regulates substrate binding and activity of BMP1 through binding to the procollagen C-propeptide trimer. Interestingly, amino acid residues that are 35 residues downstream of the BMP1 cleavage site have been reported to be critical for PCPE-1 binding and consequently for BMP1 binding and activity [69]. Striking is the fact that collagens I-III contain multiple phosphorylated sites in the PCPE-1 binding region, which might affect the binding and therefore cleavage at the C-propeptide sites (Figure 2B-E).

The N-propeptide cleavage is mediated by ADAMTS2 [70]. Three phosphorylated sites have been shown to be located 10, 11 and 15 residues downstream of the cleavage site of ADAMTS2 in procollagen α1(I). Meprin α and β have been recently reported to cleave the collagen N- and C-propeptides [71]. It is intriguing that the phosphorylated sites described above are only 4, 5, and 9 residues downstream of the N-propeptide meprin cleavage site. Phosphorylation at these sites is likely to affect the three-dimensional structure of the binding region for both ADAMTS2 and the meprins and might affect the rate of processing. In addition to the already verified phosphorylation sites, bioinformatic servers predict a large number of potential phosphorylation sites in the propeptides of collagens I, II and III, and some are located even closer to the cleavage regions. The same is true for the C-propeptide cleavage regions. One phosphorylated site is located 16 residues downstream of the BMP1 cleavage site and 7 residues downstream of the meprin α and β cleavage site in collagen α1 (I); clearly within the PCPE-1 binding region [69]. In the C-propeptide of procollagen α2(I), one experimentally verified phosphorylated site is located 5 residues upstream of the meprin α and β cleavage site and 15 residues upstream of the BMP1 cleavage site at the C-terminus. Other phosphorylated sites are located 19, 22 and 28 residues downstream of the BMP1 cleavage site, and one of these residues is known to lead to severe Osteogenesis Imperfecta type III, when mutated to P (boxed) [72]. Both propeptides of procollagen α1(II) contain verified phosphorylation sites; however, they are located at sites that are unlikely to affect the binding of ADAMTS2 or PCPE-1 and BMP1. Procollagen α1(III) contains one phosphorylated site 16 residues downstream of the BMP1 and meprin α and β C-propeptide cleavage site and many more further away.

### Phosphorylation of single collagen chains in the endoplasmic reticulum may have an effect on triple helix formation and fibril and fiber properties at a later stage

**Figure 3:**
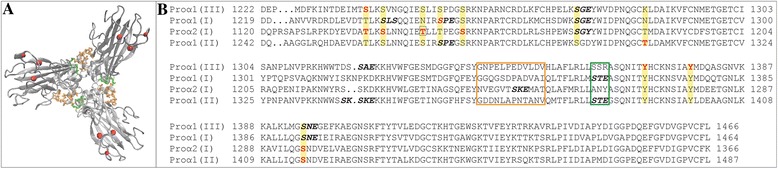
Multiple sequence alignment of the trimerization domain. **A**.: Top view of C-propeptide domain of procollagen α1(III) [74]. The discontinuous chain recognition sequence (CRS) is highlighted in orange and green and the experimentally verified phosphorylated sites in red. **B**.: Multiple sequence alignment of the C-propeptide domain of procollagen I-III. The chain recognition sequence is highlighted in an orange and green box and the experimentally verified phosphorylated sites are shown in red. The boxed residue corresponds to a known mutation leading to Osteogenesis Imperfecta [72]. Motifs recognized by FAM20C are highlighted in bold black.

Reversible protein phosphorylation plays a major role in protein synthesis, folding and secretion [73]. Several phosphorylated sites have been identified in the procollagen α1(I), α2(I), α1(II) and α1(III) chains in both the triple-helical regions and propeptide domains (Figure 1). Recently, the C-propeptide domain of procollagen α1(III) was crystallized and the structural mechanism during intracellular trimerization deciphered [74] (Figure 3A). The association of chains is governed by the chain recognition sequence (CRS), a discontinuous sequence of 15 amino acids found in procollagen α1(I), α2(I), α1(II) and α1(III). While there are no reported phosphorylated sites directly located in the chain recognition sequences, it is interesting to note that the procollagen α1(I), α2(I), α1(II) chains contain S-x-E motifs in the sequence, recognized by the FAM20C kinase which is known to be active in the Golgi apparatus [1] (Figure 3B). Another interesting fact is that the already reported phosphorylated sites are partially highly conserved; whether these have functional consequences needs to be seen. The residue T1148 in the C-propeptide region of the human procollagen α2(I) chain has been shown by mass spectrometry to be phosphorylated [72] (Figure 3B, boxed). This residue is mutated to proline in a case of severe Osteogenesis Imperfecta type III; the mutation delays triple helix formation and causes reduced secretion of the mutant molecules [72]. While replacement of threonine by proline may affect protein folding in this region, this raises the question of whether loss of a phosphorylation site may contribute to the disease phenotype. Support for a somewhat similar possibility comes from the effect of a mutation in enamelin, whereby an S216L substitution leads to loss of phosphorylation. Enamelin is phosphorylated by the FAM20C kinase at this position and the phosphorylated residue mediates calcium binding. The loss of phosphorylation at this position is regarded as the main reason for the disease Amelogenesis Imperfecta [75]. Phosphoryl groups that are attached to collagens α1(I) and α2 (I) chains inside the cell and are carried on the procollagen molecules as they are secreted into the ECM may have an effect on assembly into collagen fibrils. Given the triple-helical collagen structure, the phosphoryl groups would be located on the surface of the triple helix.

### Phosphorylation may regulate extracellular protein interactions

Phosphorylation of proteins is a common mechanism to regulate their binding to other proteins inside the cell. This mechanism may be exploited outside the cell too, as cell culture and in vitro studies show. For example, phosphorylation by PKC of T92 in the ectodomain of a membrane receptor for collagen and thrombospondin, CD36, leads to increased binding of collagen, but loss of thrombospondin binding; dephosphorylation reverses the effect [76,20]. Phosphorylation of S378 in vitronectin by PKA, adjacent to the binding sites for heparin, PAI-1 and plasminogen, reduces its binding to plasminogen and PAI-1 [77]. Phosphorylation of vitronectin at residues T50 and T57, close to the RGD integrin binding site at residues 45–47, enhances cell adhesion and accelerates cell spreading [16]. Phosphorylation of the extracellular domain of the Nogo-A receptor by CKII at the position S281 inhibits ligand binding [78].

### Collagen binding to PEDF, heparin and heparin sulfate proteoglycans

One key interacting partner of collagens is heparin. Heparin recognizes a region in collagens V and XI containing a series of conserved arginine residues [79]. Interestingly, the collagen α2(XI) chain contains a phosphorylated site in this region only 3 residues upstream and downstream of two of the conserved arginines [79] (Additional file 1: Table S1). In addition, another phosphorylated site is located in the same binding region in collagen α2(V) [79]. Phosphorylation at these residues may well affect heparin binding.

Pigment epithelium-derived factor (PEDF) is another important collagen binding partner. It has recently been discovered that the sequence IKGHRGFSGL in the C-terminal region of the human collagen α1(I) chain is a high affinity binding motif for PEDF. The motif overlaps with the heparin and heparan sulfate proteoglycan motif KGHRG(F/Y) [80]. It is interesting to note that one phosphorylated serine residue is located only 9 residues downstream of the PEDF binding sequence (Additional file 1: Table S1). PEDF itself is known to be phosphorylated extracellularly in human plasma by the kinase CKII at residues S24 and S114 and by PKA at residue S227. Phosphorylation of S24 and S114 by CKII abolishes PEDF neurotrophic activity but enhances its antiangiogenic activity; phosphorylation of S227 reduces the antiangiogenic activity [81].

### Collagen-integrin binding

Collagens contain many cell binding sites that are recognized by integrins, including RGD sites. Phosphorylation close to RGD sites may interfere with cell binding. This has already been described for collagen α3(IV). Collagen IV is a major component of basement membranes. A domain within collagen IV molecules, the Goodpasture antigen, plays an important role in the Goodpasture syndrome, a disease whereby autoimmune antibodies attack the lung and kidney. The antigen was shown to be phosphorylated in vitro at a serine residue next to an RGD sequence (*RGD***S**). In addition, the Goodpasture antigen was isolated in phosphorylated and non-phosphorylated forms from human kidney, suggesting that the phosphorylation may have a role in the disease [82]. Likewise, other collagens are reported to be phosphorylated close to the RGD site including human collagen α2(IV) at residue S1446 (**S**AVPGF*RGD*), human collagen α3(IV) at the residue S1435 (*RGD***S**), mouse collagen α2(V) at the residue S137 (**S**QGP*RGD*), human collagen α3(VI) at residue S2048 (*RGD*RGPIG**S**IG), human collagen α2(XI) at residue T576 (*RGD***T**) and human collagen α1(XXII) at residue S1038 (S*RGD*) (Additional file 1: Table S1). Phosphorylation at all these sites may well affect integrin binding.

### The role of collagen phosphorylation in bone mineralization

**Figure 4:**
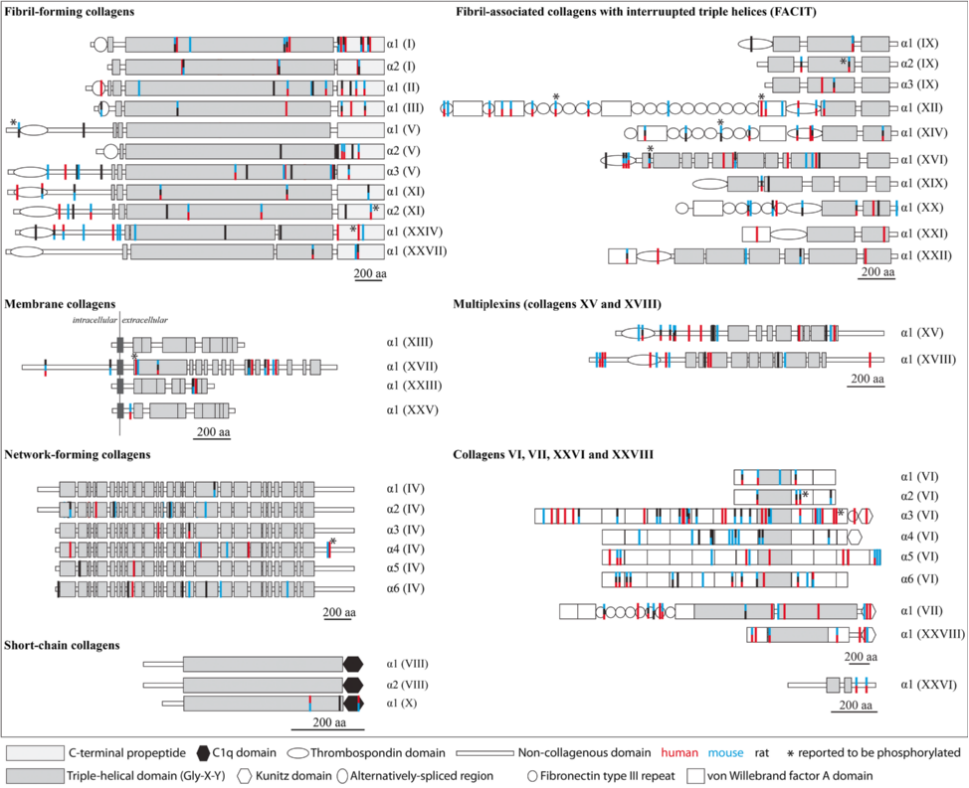
S-x-E sequence motifs in collagens. Locations of S-x-E (or S-x-Sp, whereby the second serine needs to be phosphorylated) motifs recognized by the FAM20C kinase in the collagens are highlighted [1]. The color code used for the sequence pattern S-x-E is red (human), blue (mouse) and black (rat). Reported motifs known to be phosphorylated are marked with a star. Collagen structures are represented as described [61].

Important components of the mineralized matrix of bone and dentin are secreted phosphoproteins belonging to the SIBLING family (osteopontin, bone sialoprotein, dentin sialophosphoprotein, matrix extracellular phosphoglycoprotein, dentin matrix protein 1 and many others) [83,24,84]. Phosphorylation of these proteins is important for bone mineralization; however, the stage at which they are phosphorylated is not well understood [83,85,86]. Early studies suggested that extracellular protein kinase CKII plays a key role in the mechanism [87,88]. In vitro phosphorylation of S136 in bone sialoprotein has been shown to enhance its ability to nucleate hydroxyapatite [84]. Furthermore, dephosphorylation by tartrate-resistant acid phosphatase (TRAP) is regulating osteoclast migration on phosphorylated osteopontin [89]. It has been suggested that this mechanism could control the depth and area of each osteoclastic bone resorption site by triggering osteoclast detachment and facilitating migration on the bone surface [89]. Furthermore, ectosomes have been reported to contain high concentrations of TRAP and to be released during calcification [90]. It is believed that TRAP is promoting hydroxyapatite formation and bone mineralization. While phosphorylated collagen was already reported in 1966 [91], the role of collagen phosphorylation in mineralization is less understood. As discussed above, several of the reported phosphorylated sites in the collagens are located within the sequence motif S-x-E recognized by the serine/threonine kinase FAM20C (Figure 4). The locations of many of the motifs are highly conserved among different species. Only a fraction of these motifs in collagens has been shown to be phosphorylated by mass spectrometry analyses, but even the limited data available suggest that phosphorylation in general and FAM20C phosphorylation in particular might be important for collagen functions related to biomineralization.

### Phosphorylation of matrix-associated proteolytic enzymes and growth factors such as MMPs and BMPs

#### MMPs

**Figure 5:**
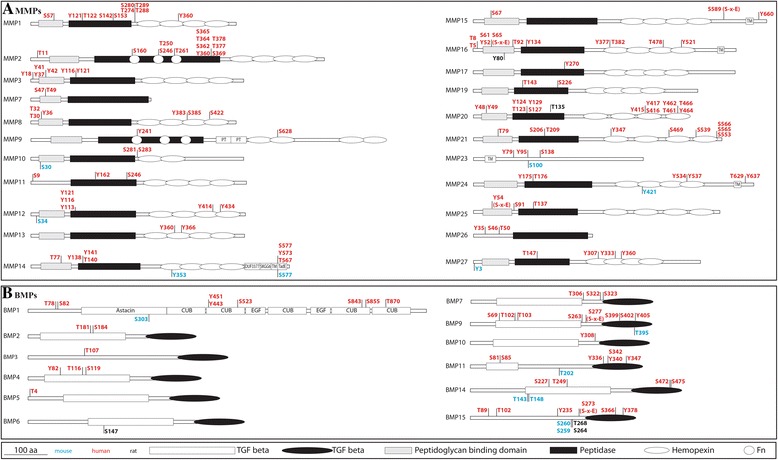
Phosphorylated residues in MMPs and BMPs. **A**.: Phosphorylated residues in MMPs. **B**.: Phosphorylated residues in BMPs. The experimentally verified phosphorylated sites were retrieved from the databases Phosida, PhosphoSitePlus, PhosphoNet, HPRD, dbPTM and UniProt. The structures are represented as used in the PhosphoSitePlus database. The color code used to represent the position of the phosphorylated sites is red (human), blue (mouse) and black (rat).

The matrix metalloproteinase MMP2 was reported to be regulated by PKC phosphorylation [92]. Human recombinant MMP2 expressed in mammalian cells was shown by mass spectrometry to be phosphorylated at residues T250, Y271 and S365. All three phosphorylated sites are located in the collagen-binding domain and the phosphorylation affects the activity of MMP2; dephosphorylation leads to increased activity, while phosphorylation diminishes the activity [92,93]. Nearly all MMPs have been reported to be partially or heavily phosphorylated, but the effects of the phosphorylation state on function remain to be studied (Figure 5A, Additional file 2: Table S2).

#### BMPs

Recombinant human BMP15, as well as growth and differentiation factor (GDF)-9, has been reported to be phosphorylated [94]. Further studies have demonstrated that BMP15 is phosphorylated by the FAM20C kinase at residue S6 and that this phosphorylation is required for biological activity [95]. In addition to these specific examples almost all bone morphogenetic proteins (BMPs), have been reported to be phosphorylated (Figure 5B, Additional file 3: Table S3). Their phosphorylation state may influence their activity. For example, BMP1 cleavage by furin at position R120 (R***S***R***S***RR-AA) leads to its activation. While there are no phosphorylated residues close to this cleavage site in the sequence, a potential site that might be recognized by FAM20C is located directly in the recognition sequence [96].

## Conclusion

Our insights into extracellular protein phosphorylation have grown during the last 40 years. With novel techniques and methods, the field has experienced a recent revival leading to astonishing and promising findings [10]. As shown in the example of collagens, several functions of extracellular matrix components may be regulated by extracellular protein phosphorylation. With the facts indicating that nearly all collagen types, MMPs and BMPs can occur in both phosphorylated and nonphosphorylated forms, we believe that phosphorylation may well represent a mechanism for regulating their functions. We speculate that extracellular protein kinases in tandem with extracellular protein phosphatases might have fundamental roles in the regulation of biochemical processes in the extracellular matrix. A better understanding of the role of extracellular protein phosphorylation of collagens and other extracellular proteins may have significant impact on cancer research, tissue engineering, regenerative medicine, biomarker development and drug design. Extracellular protein phosphorylation may affect the function of extracellular matrix, cell surface and transmembrane proteins and consequently cell behavior at large. Last but not least, there are currently a large number of experimentally verified phosphorylated sites for the proteins analyzed here. The fact that only a few sites have been shown to have functional consequences, suggests that many more examples will be discovered in the future. Furthermore, only a few of the reported sites here can be assigned to known extracellular protein kinases indicating that other protein kinases must account for the overwhelming majority of the phosphorylated sites. We thus speculate that novel extracellular protein kinases and phosphatases remain to be discovered. Their identification will open up new perspectives on the regulation of extracellular matrix functions and how to manipulate these functions in health and disease by stimulating the design of novel chemical compounds that target the extracellular matrix. Novel techniques have helped generate deep insights into the function of intracellular protein phosphorylation and led to a large number of small kinase inhibitors [97-100]. Applying such tools for studies and regulation of extracellular phosphorylation events may help decipher the functional roles of extracellular protein kinases and lead to novel and potent inhibitors for drug design.

## Additional files

Additional file 1: Table S1. Overview of all reported phosphorylated sites found for collagens. Additional file 2: Table S2. Overview of all reported phosphorylated sites found in MMPs (as of March 2015). Additional file 3: Table S3. Overview of reported phosphorylated sites found in BMPs (as of March 2015).
