# Supplementary material for: Proteomic database mining opens up avenues utilizing extracellular protein phosphorylation for novel therapeutic applications
Source: J Transl Med. 2015 Apr 19;13:125. doi: 10.1186/s12967-015-0482-4 (PMC4427915; doi:10.1186/s12967-015-0482-4)
Supplement: Additional file 1: Table S1. — Overview of all reported phosphorylated sites found for collagens. [file 12967_2015_482_MOESM1_ESM.docx]

**Additional file 1: TableS1. Overview of all reported phosphorylated sites found for collagens.**

| **Protein Species Accession number** | **Collagen binding partners** | **Phosphorylated Residue** | **All S-x-E motifs**  **Phosphorylated S-x-E** | **Known and experimentally proven poteloytic sites**  **As retrieved from PMAP-CutDB (for single references and details see database)** | **Mutations in human disease targeting S, T, Y as retrieved from UniProt**  **Phosphorylated site** |
| --- | --- | --- | --- | --- | --- |
| α1 (I)  Human: [Swiss-Prot: P02452] | **Integrin** ***R****GD* 745, 1093  **Integrin** α1β1, α1β1 596- 683-  **Heparin** 265-270 (KGHRGF) | **PhosphoSite:** S513, S522, S543, S546, S867, S889, S1023, S1029, S1125, S1247  **Phosida:** T766,  **PhosphoNet:** S171, S176, T172 | S433, S979, S991, S1247, S1271, S1359, S1393 | **MMP18 GPQG-IAGQ 775-776** | P146T (OI2, predicted p-site), G287S (OI1, predicted p-site), G353S (OI4, predicted p-site), A390T (OI2, predicted p-site), G425S (OI2, predicted p-site), G530S (OS2, 3, 4, predicted p-site), G560S (OI4, predicted p-site), G593S (OI2, 3, predicted p-site), G647S (OI1, predicted p-site), G683S (OI4, predicted p-site), G719S (OI3, predicted p-site), G722S (OI1, predicted p-site), G743S (OI2, predicted p-site), G767S (OI3, predicted p-site), **G776S (OI2**, predicted p-site**),** G809S (OI2, predicted p-site), G821S (OI3, predicted p-site), 839S (OI2, predicted p-site), G866S (OI2, 3, predicted p-site), G875S (OI2, predicted p-site), G884S (OI2, 3, predicted p-site), G906S (OI, predicted p-site), G1010S (OI4, predicted p-site), G1022S (OI3, predicted p-site), G1040S (OI2, 3, predicted p-site), G1043S (OI2, predicted p-site), G1049S (OI3, predicted p-site), G1058S (OI3, 4, predicted p-site), G1061S (OI4, predicted p-site), G1076S (OI3, predicted p-site), G1079S (OI1, OI2, predicted p-site), G1091S (OI2, predicted p-site), G1094S (OI2, predicted p-site), G1142S (OI2, predicted p-site), G1151S (OI3, predicted p-site), G1181S (OI2, predicted p-site), G1187S (OI2, 3, predicted p-site) |
| α1 (I)  Rat:  [Swiss-Prot: P02454] | **/** | **PhosphoSite:** S161, S260, S776 | S422, S968, S980, S1236, S1260, S1348, S1382 | / | / |
| α2 (I) Human: [Swiss-Prot: P08123] | **Integrin** **R**GD Site 777, 821, 1005 | **PhosphoSite:** S251, S389, S401, S404, S710, T981, T1073, S1104, T1138, S1141, **T1148,** S1155, S1295  **PhosphoNet:**S291, T306, S455 | S375, S849, S1247 | **Procollagen C-peptidase FYRA-DQPR 1119-1120** | G193S (IO4, predicted p-site), G328S (OI1, 3, 4, predicted p-site), G337S (OI3, predicted p-site), G358S (OI3, predicted p-site), G460S (OI3, predicted p-site), G592S (OI2, predicted p-site), G601S (OI, predicted p-site), 640S (OI2, predicted p-site), G733C (OI1, predicted p-site), G751S (OI4, predicted p-site), G778S (OI3, predicted p-site) ,G796S (OI2, predicted p-site), G820S (OI3, predicted p-site), G835S (OI1, predicted p-site), G949S (OI3, predicted p-site), G955S (OI2, predicted p-site), G1012S (OI3, 4, predicted p-site), **A1119T (OI**, predicted p-site**),** **T1148P (OI3**, predicted LOSS of p-site**),** C1195Y (OI, predicted p-site) |
| α2 (I)  Mouse: [Swiss-Prot: Q01149] | **Integrin R**GD Site 669, 783, 828, 1011 | **PhosphoSite:** S297, T312 | S381, S855, S1253 | / | / |
| α2 (I)  Rat:  [Swiss-Prot: P02466] | / | **PhosphoSite:** S93, S1005, S1074 | S381, S855, S1253 | / | / |
| α1 (II)  Human: [Swiss-Prot: P02458] | **Integrin** **R**GD Site 34, 137, 767, 932, 1115 | **PhosphoSite:** Y41, S536, T1307,S1416 | S77, S1001, S1193, S1271, S1341, S1382 | **MMP2, MMP9, MMP13 GAPG-NRGF 513-514**  **ADAMTS5 peptidase PGPS-GKDG 854-855**  **MMP2, MMP3, MMP8, MMP9, MMP12, MMP13 GQRG-IVGL 981-982** | C57Y (STL1O, predicted p-site), G447S (SEDC, predicted p-site), **G513S (ACG**, predicted p-site**),** G717S (ANFH, predicted p-site), G774S (SEDC, predicted p-site), **G855S (SEDC**, predicted p-site**),** G969S (ACG2, predicted p-site), **G981S (ACG2**, predicted p-site**),** G1005S (HCG, predicted p-site), G1143S (ACG2, predicted p-site), G1170S (ANFH, LCPD, predicted p-site), G1176S (SEDC, predicted p-site), G1197S (SEDC, predicted p-site), T1390N (PLSD-T, predicted LOSS of p-site), Y1391C (PLSD-T, predicted LOSS p-site), T1439M (SEDC, predicted p-site), T1448P (PLSD-T, predicted p-site) |
| α1 (III)  Human: [Swiss-Prot: P02461] | **Integrin R**GD Site 1091 | **PhosphoSite:** S1237, Y1370, Y1378 | S989, S1274, S1320, S1395 | / | G183S (EDS4, predicted p-site), G204S (EDS4, predicted p-site), G420S (EDS4, predicted p-site), G582S (EDS4, predicted p-site), G738S (EDS3, predicted p-site), G804S (EDS4, predicted p-site), G936S (EDS4, predicted p-site), G957S (EDS4, predicted p-site), G984T (EDS4, predicted p-site), G1164S (SED, predicted p-site) |
| α1 (III)  Mouse: [Swiss-Prot: P08121] | **Integrin R**GD Site 1090 | **PhosphoSite:** S783 | S39, S430, S1272, S1393 | / | / |
| α1 (III)  Rat:  [Swiss-Prot: P13941] | **Integrin R**GD Site | **PhosphoSite:** S1057 | S39, S430, S1271,S1392 | / | / |
| α1 (IV)  Human : [Swiss-Prot: P02462] | **Integrin** **R**GD Site 597, 917, 968 | **PhosphoSite:** S6, S23, Y348, S1012, S1226, T1231 | / | / | G749S (POREN1, predicted p-site) |
| α1 (IV)  Mouse:  [Swiss-Prot: P02463] | **Integrin R**GD Site 597, 781, 917, 968 | **PhosphoSite:** Y1352 | S937 | / | / |
| α2 (IV)  Human: [Swiss-Prot: P08572] | **Integrin R**GD Site 362, 517, 784, 868, 889, 970, 1069, 1228, **1452** | **PhosphoSite:**Y306, S321, S327, T1204, S1303, S1309, S1313, T1403, **S1446**, S1485, S1683, S1687  **PhosphoNet:** Y1586, Y274 | S310 | **/** | A1690T (HS, predicted p-site) |
| α2 (IV)  Mouse: [Swiss-Prot: P08122] | **Integrin R**GD Site 141, 360, 779, 884, 965, 1223, 1447 | **PhosphoSite:**Y182 | S175, S410, S708, S890 | / | / |
| α3 (IV)  Human: [Swiss-Prot: Q01955] | **Integrin** **R**GD Site 791, 996, 1154, 1306, 1345, **1432** | **PhosphoSite:**T235, T243, T249, S382, T835, S1246, S1249, **S1435**, T1443,S1452, S1648, T1649 | S589, S764 | **/** | G1277S (APSAR, predicted p-site), I1330T (APSAR, predicted p-site) |
| α3 (IV)  Mouse: [Swiss-Prot: Q9QZS0] | **Integrin R**GD Site 830, 994, 1152, 1304 | **PhosphoSite:** S1575 | ***/*** | / | / |
| α4 (IV)  **Human:** [Swiss-Prot: **P53420]** | **Integrin R**GD Site 94, 145, 189, 310, 724, 785, 989, 1212 | **PhosphoSite:** S139, S1173, S1283,S1425 S1662, S1663  **PhosphoNet:** S1540, S1545,**S1555,** Y1541,Y1562 S562 | S88, S827, S1102, **S1555** | / | A931T (APS, predicted p-site), G1201S (APSAR, predicted LOSS of p-site), P1402S (APS, predicted LOSS of p-site) |
| α4 (IV)  Mouse: [Swiss-Prot: Q9QZR9] | **Integrin R**GD Site 86, 137, 181, 587, 593, 716, 980, 992, 1144 | **PhosphoSite:** S554 | S819, S977, S1547 | / |  |
| α5 (IV)  Human: [Swiss-Prot: P29400] | / | **PhosphoSite:** S33, S962, S1343,  **HPRD:** S543 | S447 | / | G114S (APSX, predicted p-site), G219S (APSX, predicted LOSS of p-site), G298S (APSX, predicted p-site), G521S (APSX predicted p-site), P619S (APSX, predicted LOSS of p-site), G638S (APSX, predicted p-site), P739S (APSX, predicted p-site), G1030S (APSX, predicted p-site), G1039S (APSX, predicted p-site), G1066S (APSX, NOT PREDICTED), G1143S (APSX, predicted p-site), G1167S (APSX, predicted p-site), G1170S (APSX, predicted p-site), G1252S (APSX, predicted p-site), G1270S (APSX, predicted p-site), G1333S (APSX, predicted p-site), G1357S (APSX, predicted p-site), G1451S (APSX, predicted p-site), S1488F (APSX, predicted LOSS of p-site), P1517T (APSX, predicted p-site), W1538S (APSX, predicted p-site), C1564S (APSX, predicted p-site) |
| α6 (IV)  Human: [Swiss-Prot: Q14031] | **Integrin R**GD Site 515, 560, 986 | **PhosphoSite:** T686, S802, T805, S963, S979, S1047, S1049,T1335 | S442 | / | / |
| α1 (V)  Human: [Swiss-Prot: P20908] | **Integrin** **R**GD Site 645, 663  **Heparin** 898 - 927 | **PhosphoSite:**T64, Y442, T567, S884, S1314, S1317 S1702  **Phosida:** T957  **PhosphoNet:** S68 | / | / | G530S (EDS1, 2, predicted p-site), C1639S (EDS1, predicted p-site) |
| α1 (V)  Mouse: [Swiss-Prot: O88207] | **Integrin R**GD Site 645, 663 | **PhosphoSite:S68,** T699, Y788 | **S68** | / | / |
| α2 (V)  Human: [Swiss-Prot: P05997] | **Integrin R**GD Site 506, 944, 1067, 1070, 1100, 1127, 1136  **Heparin** 555-584 | **PhosphoSite:** T326, S376, T575, T790, T901, T908, S913, T988, T989 S1276, S1277 T1281, S1284  **PhosphoNet:** S139 | S1308, S1319, S1394 | / | / |
| α3 (V)  **Human:** [Swiss-Prot: **P25940]** | **Integrin R**GD Site 565, 1207, 1474  **Heparin** 818 - 847 | **PhosphoSite:** Y110, S718, S1315, T1684, T1685 | S279, S1072, S1300, S1524, S1601 | / | / |
| α1 (VI)  Human: [Swiss-Prot: P12109] | **Integrin R**GD Site 262, 442, 478 | **PhosphoSite:** T104, Y152, T157, Y165, S348, Y793, Y878, S1008  **Phosida:** S188  **PhosphoNet:** Y527, Y542, | S94, S249, S622 | / | S116N (BM, predicted LOSS of p-site), K571T (BM, predicted p-site), T881M (BM, predicted LOSS of p-site) |
| α2 (VI)  Human: [Swiss-Prot: P12110] | **Integrin** **R**GD Site 366, 426, 489, 498, 539 | **PhosphoSite:**S126, S127, S399, Y597, Y665, **S666,** S683, S685, T701, T703, S705 | S223, S622, **S666** | / | G271S (BM, predicted p-site), P518S (BM, predicted p-site), G700S (BM, predicted p-site), R876S (UCMD, predicted p-site), S895R (BM, predicted LOSS of p-site) |
| α3 (VI)  Human: [Swiss-Prot: P12111] | **Integrin R**GD Site **2040**, 2136, 2148, 2154, 2370 | **PhosphoSite:**T115, S354, T433, T510, Y524, Y543, S934, Y1072, S1225, Y1318, S1320, T1409, T1410, S1471, Y1493, S1512, T1929, **S2048,** S2492, T2498, Y2767, **S2821*,*** Y2827, S2829, S3079, Y3142 | S162, S222, S287, S359, S1025, S1384, S1399, S1443, S1755, S1783, S2127, S2164, S2486, S2626, S2694, S2801, **S2821,** S2992, S3100 | / | A807T (BM, predicted p-site), A830S (BM, predicted p-site) |
| α3 (VI)  Mouse: [Swiss-Prot: O88493] | **Integrin R**GD Site 1430, 1526, 1538, 1544, 1760 | **PhosphoSite:**Y471, S1173, Y1353, T1714, S1862, S1876 | S86, S415, S774, S1145, S1173, S1356, S1427, S1445, S1517, S1876, S1935, S2016, S2191, S2211, S2481, S2643, S2652 | / | / |
| α3 (VI)  Rat:  [Swiss-Prot: D4A115]  [Swiss-Prot: NP_001102478] | **Integrin R**GD Site 1429, 1525, 1537, 1543, 1759 | **PhosphoSite:** S1780 | S415, S774, S1144, S1172, S1355, S1426, S1444, S1516, S1875, S1934, S2015, S2190 | / | / |
| α5 (VI)  Human: [Swiss-Prot: A8TX70] | **Integrin** **R**GD Site 1430 | **PhosphoSite:** T51, S59, Y67, S228, S280, S306, S312, T314, S386, Y387, S419, S422, T423, Y424, S1411, S2414, T2418 | S75, S143, S173, S1350, S1566, S2255, S2304, S2577 | / | / |
| α5 (VI)  Mouse: [Swiss-Prot: A6H584] | **Integrin R**GD Site 1649, 2216, 2259 | **PhosphoSite:** Y2372 | S143, S173, S1027, S1382, S2578, S2610, S2623 | / | / |
| α6 (VI)  Human: [Swiss-Prot: A6NMZ7] | **Integrin R**GD Site 1508 | **PhosphoSite:** Y247, S321, S371,T412, T414, S416, S513, T515, S629, S632, T730, Y966, S1369, S1489, S1492, Y1833 | S140, S170, S241, S274, S1228, S1317, S1519, S1839, S1978 | / | / |
| α6 (VI)  Rat: [Swiss-Prot: XP_001073177] | / | **PhosphoSite:** Y965 | S139, S169, S240, S273, S666, S712, S1227, S316, S1453 | / | / |
| α1 (VII)  Human: [Swiss-Prot: Q02388] | **Integrin** **R**GD Site 1170, 1334, 2008, 2553 | **PhosphoSite:** Y35, S47, T117, S202, T354, Y356, S402, T597, T691, T695, T703, T1318, S1327, S1686, S1690 | S416, S732, S828, S945, S959, S1984, S2104, S2421, S2859, S2900 | / | T119P (BC, predicted LOSS of p-site), P1364T (BC, predicted p-site), G2031S (RDEB, predicted p-site), G2040S (DEB, predicted p-site), G2192S (RDEB, predicted p-site), G2366S (RDEB, predicted p-site), G2369S (EBP, predicted p-site), G2775S (RDEB, predicted p-site) |
| α1 (VII)  Mouse: [Swiss-Prot: Q63870] | **Integrin R**GD Site 1171, 2002, 2063, 2601, 2631 | **PhosphoSite:** T1017, S2264, S2270 | S417, S829, S879, S960, S1736, S2045, S2851 | / | / |
| α1 (VIII)  Human: [Swiss-Prot: P27658] | / | **PhosphoSite:**Y613  **PhosphoNet:** S20, S21 | / | / | / |
| α1 (VIII)  Mouse: [Swiss-Prot: Q00780] | / | **PhosphoSite:**S18 | / | / | / |
| α2 (VIII)  Human: [Swiss-Prot: P25067] | **Integrin R**GD Site 149, 393 | **PhosphoSite:**Y648 | S687 | / | / |
| α2 (VIII)  Mouse: [Swiss-Prot: P25318] | **Integrin R**GD Site 145, 195, 389 | **PhosphoSite:**T535 | S683 | / | / |
| α2 (IX)  Human: [Swiss-Prot: Q14055] | / | **PhosphoSite:**S7, S10, **S459** | S219, **S459** | / | / |
| α2 (IX)  Mouse: [Swiss-Prot: Q07643] | / | **PhosphoSite:**S676 | S218, S458 | / | / |
| α3 (IX)  Human: [Swiss-Prot: Q14050] | **Integrin R**GD Site 423, 601 | **PhosphoSite:**T290, S297, S534 | S297, S363 | / | / |
| α1 (X)  Mouse: [Swiss-Prot: Q05306] | / | **PhosphoSite:**T164 | S501, S664 | / | / |
| α1 (XI)  Human: [Swiss-Prot: P12107] | **Heparin** 868-885 | **PhosphoSite:** T57, T58, T62, S67, S70, T72, Y74, S193, S355, Y386, S1682, Y1721, T1766, T1772  **PhosphoNet:**Y317 | S50, S170, S741, S1350 | / | / |
| α2 (XI)  Human:  [Swiss-Prot: P13942] | **Integrin R**GD Site **573**, 591, 1401  **Heparin** 826-855 | **PhosphoSite:**S42, Y60, S506, **T576**, S686, T701, T777, T843, S1439, S1519 **S1675**, T1678, S1679, Y1681 | S220, S365, S822, S1164, **S1675** |  | P621T (DFNB53 predicted p-site) |
| α1 (XII)  Human: [Swiss-Prot: Q99715] | **Integrin R**GD Site 862, 2779, 2895 | **PhosphoSite:** T67, T217, S273, **S765**, T1082, S1261, T1292, S1312, T1397, S1433, S1438, Y1450, T1743, **S2125**, T2231, T2243, T2433, S2437,  S2536, S2541,S2861, S2864,S2952, S2994, Y3043, S3062  **Phosida:** S97, S977, S1452, S1495, S1788, T549, S2141, S2616, S2659  **PhosphoNet:**T509, Y2667 | S20, S329, S412, S469, S609, **S765**, S981, **S2125**, S2155, S2369, S2488, S2541 | / | / |
| α1 (XII)  Mouse: [Swiss-Prot: Q60847] | **Integrin R**GD Site 862, 2781, 2897 | **PhosphoSite:** S353, S381, S1101, S1102, T1106, Y2669 | S20, S44, S329, S412, S469, S609, S765, S981, S1512, S2157, S2265, S2371, S2490, S2543 | / | / |
| α1 (XII)  Rat:  [Swiss-Prot: P70560] | **Integrin R**GD Site 94 | **PhosphoSite:** S647, S661 | / | / | / |
| α1 (XIII)  Human: [Swiss-Prot: Q5TAT6] | / | **PhosphoSite:** S245 | / | / | / |
| α1 (XIV)  Human: [Swiss-Prot: Q05707] | **Integrin R**GD Site 1607 | **PhosphoSite:** T439, Y662, S963, T970, Y988, T989, Y1118, T1125, S1128, S1648, Y1758, Y1767 | S143, S838, S1154, S1245, S1250, S1721 | / | / |
| α1 (XIV)  Mouse: [Swiss-Prot: Q80X19] | **Integrin R**GD Site 1608 | **PhosphoSite:** **S649** | S144, S416, **S649**, S839, S1155, S1251, S1722 | / | / |
| α1 (XIV)  Rat: [Swiss-Prot: NP_001124020] | / | **PhosphoSite:** S649, S1726, S1732 | S144, S416, S649, S836, S1152, S1248, S1719 | / | / |
| α1 (XV)  Human: [Swiss-Prot: P39059] | / | **PhosphoSite:** S976, T1136, S1139, T1344, T1350 | S146, S243, S293, S319, S390, S454, S976, S1162 | / | / |
| α1 (XV)  Mouse: [Swiss-Prot: O35206] | / | **PhosphoSite:** Y137 | S126, S146, S243, S293, S313, S318, S541, S1141 | / | / |
| α1 (XV)  Rat: [Swiss-Prot: NP001094005]  [Swiss-Prot: Q4G024] | / | **PhosphoSite:** S66, S67, T77, Y85, | S146, S243, S293, S313, S318, S519, S523, S1119 | / | / |
| α1 (XVI)  Human: [Swiss-Prot: Q07092] | **Integrin** **R**GD Site 540, 1006, 1227 | **PhosphoSite:** S4, T17, T105, S612, T855, Y1361, S1429, Y1436, S1492  **PhosphoNet:** Y1109 | S132, S137, S141, S260, S655, S705, S996, S1146 | / | / |
| α1 (XVI)  Mouse: [Swiss-Prot: Q8BLX7] | **Integrin** **R**GD Site 555, 1000, 1206 | **PhosphoSite:** T258, **S260** | S132, S137, S141, **S260,** S714, S1092, S1125 | / | / |
| α1 (XVI)  Rat:  [Swiss-Prot: F1LND0] | / | **PhosphoSite:** S1213, S1428, S1506 | S31, S132, S137, S260, S711, S1160 | / | / |
| α1 (XVII)  Human: [Swiss-Prot: Q9UMD9] | / | **PhosphoSite:** Y40, S56, S61, S62, Y64, S67, S70, S79, Y80, S85, S88, S93, S96, T105, Y109, S114, S148, S150, S152, T153, T156, S172, S174, T182, T303, Y305, S384, Y396, S400, T512, S515, S542, **S544**, S640, S1124, S1131, T1284, S1287, S1296, S1299, S1300, Y1395  **PhosphoNet:**S170 | S118, **S544**, S640, S1083, S1160, S1197  (6) | / | S265C (GABEB predicted LOSS of p-site) |
| α1 (XVII)  Mouse: [Swiss-Prot: Q07563] | **Integrin R**GD Site 695 | **PhosphoSite:** S85, S88, S93, S149, S151, S171, S175, S380 | S119, S422, S551, S647, S1070, S1147, S1184 | / | / |
| α1 (XVII)  Rat: [Swiss-Prot: D3ZSH7] | / | **PhosphoSite:** S85, S93, S427 | S119, S422, S647, S1070, S1147 | / | / |
| α1 (XVIII) Human: [Swiss-Prot: P39060] | **Integrin R**GD Site 1330 | **PhosphoSite:** T93, S99, T696, T697, S704, S705, S706, S709, S711, Y1211, S1245, T1450 | S70, S79, S96, S362, S454, S705, S711, S1653 | / | / |
| α1 (XIX)  Human: [Swiss-Prot: Q14993] | **Integrin R**GD Site 952 | **PhosphoSite:** Y46, S81, S184, T487, Y722, S724, Y829, S1093, T1099, S1100  **PhosphoNet:** Y1130 | S1014 | / | / |
| α1 (XIX)  Mouse: [Swiss-Prot: Q0VF58] | **Integrin R**GD Site 946 | **PhosphoSite:** Y1124 | S1008 | / | / |
| α1 (XX)  Human: [Swiss-Prot: Q9P218] | / | **PhosphoSite:** S52, Y56, T74, S545, T554, T556, S594, S599, T602, T609, S610, S617, S618, S619, T639, T643, T644, Y856, T1172 | S430, S593, S1067, S1164 | / | / |
| α1 (XXII)  Human: [Swiss-Prot: Q8NFW1] | **Integrin R**GD Site 969, **1039**, 1395 | **PhosphoSite:** S34, Y37, T45,Y110, T117, S130, S253, S534, S930, S1035, **S1038**,T1281, S1284, S1293 | S97, S280, S1460 | / | / |
| α1 (XXII)  Mouse:  [Swiss-Prot: E9Q7P1] | **Integrin R**GD Site 976, 1026, 1382 | **PhosphoSite:** S199 | S106, S780, S1089 | / | / |
| α1 (XXII)  Rat:  [Swiss-Prot: F1M897  XP_243609] | / | **PhosphoSite:** S955, S1008 | S106, S1091 | / | / |
| α1 (XXIII)  Human: [Swiss-Prot: Q86Y22] | **Integrin R**GD Site 132, 174 | **PhosphoSite:** T248, S450, T467 | S444 | / | / |
| α1 (XXIV)  Human: [Swiss-Prot: Q17RW2] | / | **PhosphoSite:** T328, Y454, Y472, Y473, Y474, T962, S982, T983, T1033, Y1295, S1302, T1537, **S1611**, T1625 | S187, S227, S464, S1514, **S1611** | / | / |
| α1 (XXIV)  Mouse: [Swiss-Prot: Q30D77] | / | **PhosphoSite:** S938 | S189, S233, S326, S483, S493, S567, S1630 | / | / |
| α1 (XXV)  Human:  [Swiss-Prot: Q9BXS0] | / | **PhosphoSite:** T30, S468, Y585 | S101 | / | / |
| α1 (XXVII)  **Human:** [Swiss-Prot: **Q8IZC6]** | **Integrin R**GD Site 1206, 1354, 1594 | **PhosphoSite:** S419, T456, T460, S539, S542, S1028, T1040, S1052, S1582  **PhosphoNet:** S373,S549, S550, S576, | S1531, S1756 | / | / |
| α1 (XXVIII)  Human:  [Swiss-Prot: Q2UY09] | **Integrin R**GD Site 741 | **PhosphoSite:** T692 | S55, S86, S207, S805, S1004, S1049 | / | / |

The databases Phosida (http://www.phosida.com/), PhosphoSitePlus (http://www.phosphosite.org), PhosphoNet (http://www.phosphonet.ca/), HPRD (http://www.hprd.org/) and UniProt (http://www.uniprot.org/) were searched for all human, mouse and rat collagens. For additional information to the single detected site refer to their entries in the databases. The following shortcuts are used in the table: Schmid Metaphyseal Chondrodysplasia (SMCD), Atrophic Benign Epidermolysis Bullosa (ABEB), Autosomal Recessive Non-syndromic Hearing Loss (DFNB53), Epidermolysis Bullosa Pruriginosa (EBP), Dystrophic Epidermolysis Bullosa (DEB), Breast Cancer (BC), Ullrich Congenital Muscular Dystrophy (UCMD), Bethlem Myopathy (BM), X-linked Alport Syndrome (APSX), Autosomal Alport Syndrome (APSAR), impair COL4A1 and COL4A2 secretion and cause hemorrhagic stroke (HS), Perinatal Cerebral Hemorrhage and Porencephaly (POREN1), Spondyloepiphyseal Dysplasia (SED), Colorectal Cancer (CC), Ehlers Danlos Syndrome (EDS), Platyspondylic Lethal Skeletal Dysplasia (PLSD), Legg-Calve-Perthes Disease (LCPD), Hypochondrogenesis (HCG), Inherited Osteonecrosis of Femoral Head (ANFH), Achondrogenesis, Spondyloepiphyseal Dysplasia (SEDC), Ocular Variant Stickler Syndrome (STL1O), Osteogenesis Imperfecta (OI). **Procedure:** The UniProt database (http://www.uniprot.org/) was searched for the protein accession number of the human protein (*e.g. P02452*). The link to the PhosphoSite database in the UniProt database was used to obtain the entries for each single protein. Due to constant updates and modifications of the criteria for a database entry in the PhosphoSite database, the data presented here may slightly vary from the database entries in the future (Hornbeck et al. 2014, Nucleic Acids Research, doi: 10.1093/nar/gku1267). The Phosida and HPRD database were searched for protein accession numbers.
