# Supplementary material for: Proteomic database mining opens up avenues utilizing extracellular protein phosphorylation for novel therapeutic applications
Source: J Transl Med. 2015 Apr 19;13:125. doi: 10.1186/s12967-015-0482-4 (PMC4427915; doi:10.1186/s12967-015-0482-4)
Supplement: Additional file 2: Table S2. — Overview of all reported phosphorylated sites found in MMPs (as of March 2015). [file 12967_2015_482_MOESM2_ESM.docx]

**Additional file 2: Table S2. Overview of all reported phosphorylated sites found in MMPs (as of March 2015).**

| **Protein**  **Species**  **Accession number** | **Phosphorylated residues as retrieved from the PhosphositePlus database** | **All S-x-E motifs**  ***Phosphorylated S-x-E*** | **Mutations in human diseases targeting the residues S, T, Y as retrieved from UniProt** |
| --- | --- | --- | --- |
| MMP1 Human: [Swiss-Prot: P03956] | S57, Y121, T122, S142, S153, T274, S280, T288, T289, Y360 | S382 | / |
| MMP2 Human: [Swiss-Prot: P08253] | T11, S160, S246, T250, T261, Y360, S362, T364, S365, S369, T377, T378 | / | D210Y (osteolysis and arthritis syndrome) A228T (colorectal cancer) T498M (colorectal cancer) S644I (colorectal cancer) |
| MMP3 Human: [Swiss-Prot: P08254] | Y18, Y37, Y41, Y42, Y116 , Y121 | S269, S417 | / |
| MMP7 Human: [Swiss-Prot: P09237] | S47, T49 | S54 | / |
| MMP8 Human: [Swiss-Prot: P22894] | T30, T32, Y36, Y383, S385, S422 | S25 | / |
| MMP9 Human: [Swiss-Prot: P14780] | Y241, S628 | / | / |
| MMP10 Human: [Swiss-Prot: P09238] | S281, S283 | S268, S416 | / |
| MMP10 Mouse: [Swiss-Prot: O55123] | S30 | / | / |
| MMP11 Human: [Swiss-Prot: P24347] | S9, Y162, S246 | / | / |
| MMP12 Human: [Swiss-Prot: P39900] | Y113, Y116, Y121, Y414, Y434 | S24 | / |
| MMP12 Mouse: [Swiss-Prot: P34960] | S34 | / | / |
| MMP13 Human: [Swiss-Prot: P45452] | Y360, Y366 | S32 | F74S (metaphyseal anadysplasia)  F75S (spondyloepimetaphyseal dysplasia, Missouri type) in SEMD-MO; abnormal intracellular autoactivation and autodegradation within the ER/Golgi resulting in the secretion of small and inactive fragments  M91T (metaphyseal anadysplasia) |
| MMP14 Human: [Swiss-Prot: P50281] | T77, Y138, T140, Y141, T567, Y573, S577 | S34, S217, S394, S470 | / |
| MMP14 Mouse: [Swiss-Prot: P53690] | Y353, S577 | S34, S217, S394, S470 | / |
| MMP15 Human: [Swiss-Prot: P51511] | S67, ***S589,*** Y660 | S160, ***S589*** | / |
| MMP16 Human: [Swiss-Prot: P51512] | T5, T8, Y52, S61, ***S65***, T92, Y134, Y377, T382, T478, Y521 | ***S65,*** S224, S463 | / |
| MMP16 Rat: [Swiss-Prot: O35548] | Y80 | S65, S224, S463 | / |
| MMP17 Human: [Swiss-Prot: ]Q9ULZ9 | Y270 | / | / |
| MMP19 Human: [Swiss-Prot: Q99542] | T143, S226 | S290 | / |
| MMP20 Human: [Swiss-Prot: O60882] | Y48, Y49, T123, Y124, S127, Y129, Y415, S416, Y417, T461, Y462, Y464, T466 | S135, S162, S170 | / |
| MMP20 Rat: [Swiss-Prot: D3ZXD3] | T135 | S161, S169 | / |
| MMP21 Human: [Swiss-Prot: Q8N119] | T79, S206, T209, Y347, S469, S539, S553, S565, T566 | S72, S101, S328 | / |
| MMP23 Human: [Swiss-Prot: O75900] | Y79, Y95, S138 | / | / |
| MMP23 Mouse: [Swiss-Prot: O88676] | S100 | S107 | / |
| MMP24 Human: [Swiss-Prot: Q9Y5R2] | Y175, T176, Y534, Y537, T629, Y637 | S260, S500, S530, S553 | / |
| MMP24 Mouse: [Swiss-Prot: Q9R0S2] | Y421 | S233, S473, S503, S526 | / |
| MMP25 Human: [Swiss-Prot: Q9NPA2 | **S54**, S91, T137 | **S54**, S134 | / |
| MMP26 Human: [Swiss-Prot: Q9NRE1 | Y35, S46, T50 | / | / |
| MMP27 Human: [Swiss-Prot: Q9H306 | T147, Y307, Y333, Y360 | S42 | / |
| MMP27 Mouse: [Swiss-Prot: D3ZQ07 | Y3 | S42, S477 | / |

The data were retrieved from the PhosphoSitePlus database (http://www.phosphosite.org; PubMed: 15174125) and the UniProt database (http://www.uniprot.org/; PMID: 23161681). For more information to the specific phosphorylated sites (method and samples used) please refer to the database entries. The S-x-E motifs were obtained by sequence analysis. **Procedure:** The UniProt database (http://www.uniprot.org/) was searched for the protein accession number (*e.g. P02452*). The link to the PhosphoSite database in the UniProt database was used to obtain the entries for each single protein. Due to constant updates and modifications of the criteria for a database entry, the data presented here may slightly vary from the database entries in the future (Hornbeck et al. 2014, Nucleic Acids Research, doi: 10.1093/nar/gku1267).
