# Supplementary material for: Proteomic database mining opens up avenues utilizing extracellular protein phosphorylation for novel therapeutic applications
Source: J Transl Med. 2015 Apr 19;13:125. doi: 10.1186/s12967-015-0482-4 (PMC4427915; doi:10.1186/s12967-015-0482-4)
Supplement: Additional file 3: Table S3. — Overview of reported phosphorylated sites found in BMPs (as of March 2015). [file 12967_2015_482_MOESM3_ESM.docx]

**Additional file 3: Table S3. Overview of reported phosphorylated sites found in BMPs (as of March 2015).**

| **Protein**  **Species**  **Accession number** | **Phosphorylated residue as retrieved from the PhosphositePlus database** | **All S-x-E motifs**  **Phosphorylated S-x-E** | **Mutations in human disease targeting S, T, Y as retrieved from UniProt** |
| --- | --- | --- | --- |
| BMP1 Human: [Swiss-Prot: P13497 | T78, S82, Y443, Y451, S523, S843, S855, T870 | S335, S479, S724 | / |
| BMP1 Mouse: [Swiss-Prot: P98063 | S303 | S340, S484, S729, S862 | / |
| BMP2 Human: [Swiss-Prot: P12643 | T181, S184 | S46, S117, S147 | / |
| BMP3 Human: [Swiss-Prot: P12645 | T107 | S78, S282 | / |
| BMP4 Human: [Swiss-Prot: P12644 | Y82, T116, S119 | S50, S91, S155 | S91C (renal hypodysplasia) |
| BMP5 Human: [Swiss-Prot: P22003 | T4 | / | / |
| BMP6 Rat: [Swiss-Prot: Q04906 | S147 | S294, S398 | / |
| BMP7 Human: [Swiss-Prot: P18075 | T306, S322, S323 | S48, S219, S248 | / |
| BMP9 Human: [Swiss-Prot: Q9UK05 | S69, T102, T103, S263, **S277**, S399, S402, Y405 | S113, **S277**, S292, S299 | / |
| BMP9 Mouse: [Swiss-Prot: Q9WV56 | T395 | S112, S297 | / |
| BMP10 Human: [Swiss-Prot: O95393 | Y308 | S188, S275 | / |
| BMP11 Human: [Swiss-Prot: O95390 | S81, S85, Y336, Y340, S342, Y347 | S71, S96, S308 | / |
| BMP11 Mouse: [Swiss-Prot: Q9Z1W4 | T202 | S69, S94, S306 | / |
| BMP14 Human: [Swiss-Prot: P43026 | S227, T249, S472, S475 | / | C400Y (AMDG) P436T (DPS) S439T (DPS) S475N (SYNS2) |
| BMP14 Mouse: [Swiss-Prot: P43027 | T143, T148 | / | / |
| BMP15 Human: [Swiss-Prot: O95972 | T89, T102, Y235, **S273**, S366, Y378 | S166, **S273** | Y235C (ODG2) A180T (POF4) |
| BMP15 Mouse: [Swiss-Prot: Q9Z0L4 | S259, S260 | S271, S280 | / |
| BMP15 Rat: [Swiss-Prot: Q9WUW1 | S264, T268 | S121, S279 | / |

The data were retrieved from the PhosphoSitePlus database (http://www.phosphosite.org; PubMed: 15174125) and the UniProt database (http://www.uniprot.org/; PMID: 23161681). For more information to the specific phosphorylated sites (method and samples used) refer to the database entries. The S-x-E motifs were obtained by sequence analysis. Following shortcuts are used in the table: Acromesomelic Chondrodysplasia, Grebe type (AMDG), Du Pan Syndrome (DPS), Multiple Snostoses Syndrome 2 (SYNS2), Ovarian Dysgenesis 2 (ODG2) and Premature Ovarian Failure 4 (POF4). **Procedure:** The UniProt database (http://www.uniprot.org/) was searched for the protein accession number (*e.g. P02452*). The link to the PhosphoSite database in the UniProt database was used to obtain the entries for each single protein. Due to constant updates and modifications of the criteria for a database entry, the data presented here may slightly vary from the database entries in the future (Hornbeck et al. 2014, Nucleic Acids Research, doi: 10.1093/nar/gku1267).
